# Clinical Significance of ABCB1 in Acute Myeloid Leukemia: A Comprehensive Study

**DOI:** 10.3390/cancers11091323

**Published:** 2019-09-06

**Authors:** Thomas Boyer, Fanny Gonzales, Adeline Barthélémy, Alice Marceau-Renaut, Pauline Peyrouze, Soizic Guihard, Pascale Lepelley, Adriana Plesa, Olivier Nibourel, Carole Delattre, Marc Wetterwald, Nicolas Pottier, Isabelle Plantier, Stéphane de Botton, Hervé Dombret, Céline Berthon, Claude Preudhomme, Christophe Roumier, Meyling Cheok

**Affiliations:** 1 Laboratory of Hematology, CHU Lille, 59000 Lille, France; 2 UMR-S 1172, Jean-Pierre AUBERT Research Centre, University Lille, Inserm, CHU Lille, 59000 Lille, France; 3 Laboratory of Hematology, Hospital of Lyon-South, 69495 Pierre–Benite, France; 4 Laboratory of Hematology, Hospital of Dunkerque, 59240 Dunkerque, France; 5 Department of Hematological Diseases, Hospital of Dunkerque, 59240 Dunkerque, France; 6 Department of Biochemistry, University Hospital Lille, 59000 Lille, France; 7 Department of Hematological Diseases, Hospital of Roubaix, 59100 Roubaix, France; 8 Department of Clinical Hematology, Gustave Roussy Institute, 94800 Paris, France; 9 Department of Hematology, University Paris 7, 75013 Paris, France; 10 Department of Hematological Diseases, University Hospital of Lille, 59000 Lille, France

**Keywords:** acute myeloid leukemia, ABCB1, drug resistance, gene expression, prognosis

## Abstract

ABCB1 is a member of the ATP binding cassette transporter family and high ABCB1 activity is considered as a poor prognostic factor in acute myeloid leukemia (AML) treated with intensive chemotherapy, its direct relation with drug resistance remains unclear. We evaluated ABCB1 activity in relation with clinical parameters and treatment response to standard chemotherapy in 321 patients with de novo AML. We assessed multiple clinical relationships of ABCB1 activity—ex vivo drug resistance, gene expression, and the ABCB1 inhibitor quinine were evaluated. ABCB1 activity was observed in 58% of AML and was linked to low white blood cell count, high expression of CD34, absence of *FLT3-ITD*, and absence of mutant *NPM1*. Moreover, ABCB1 activity was associated with worse overall- and event-free survival. However, ABCB1 activity did not directly lead to ex vivo drug resistance to anthracyclines. We found that *ABCB1* was highly correlated with gene expressions of *BAALC, CD34, CD200*, and *CD7*, indicating that *ABCB1* expression maybe a passenger characteristic of high-risk AML. Furthermore, *ABCB1* was inversely correlated to *HOX* cluster genes and *CD33*. Thus, low *ABCB1* AML patients benefited specifically from anti-CD33 treatment by gemtuzumab ozogamicin in addition to standard chemotherapy. We showed prognostic importance of ABCB1 gene expression, protein expression, and activity. Furthermore, ABCB1 was not directly linked to drug resistance, ABCB1 inhibition did not improve outcome of high ABCB1 AML patients and thus high ABCB1 may represent a passenger characteristic of high-risk AML.

## 1. Introduction

Acute myeloid leukemia (AML) is a heterogeneous disease and chemotherapy resistance is the major cause of leukemia related deaths [1]. During the past few decades, multiple genetic alterations and mutations have been described and the most relevant of them were included into the European Leukemia Net (ELN) recommendations for diagnosis and management of AML [2]. Regardless of known molecular alterations, unexplained treatment failures still occur due to anti-leukemia drug resistance. One plausible candidate conferring drug resistance are the members of the ATP binding cassette (ABC) transporter superfamily, which affect drug disposition by exporting a variety of chemotherapeutics out of the target cell [3]. In particular, ABC subfamily B-member 1 (ABCB1) also known as permeability glycoprotein and multi-drug resistance (P-gp or MDR1), has been extensively studied in solid cancers and leukemia [4]. Indeed, ABCB1 may induce resistance to anthracyclines, a cornerstone class of drugs used as first-line AML therapy in most current treatment protocols world-wide. Approximately 50% of AML patients have blast cells that express ABCB1 [5] and over-expression of ABCB1 was associated with lower complete remission (CR) rates and higher relapse rates in patients treated with standard chemotherapy based on the combination of an anthracycline with cytarabine [5,6,7,8]. AML blasts expressing ABCB1 showed reduced intracellular daunorubicin levels after ex vivo drug exposure [9]. Idarubicin is an alternate anthracyclin which has been proposed for patients expressing high levels of ABCB1 [10]. Similarly, higher ABCB1 activity and lower CD33 expression was related to lower response rates in AML patients treated with gemtuzumab ozogamicin (GO), an immunoconjugate of an anti-CD33 antibody with a toxic calicheamicin-γ derivative [11]. Thus, ABCB1 expression may be useful in clinical routine diagnostics to optimize chemotherapy selection. Furthermore, competitive inhibitors of ABCB1 such as quinine or cyclosporine A have been shown to reverse the MDR phenotype [12], and combination thereof with chemotherapy in high-risk AML with high ABCB1 activity resulted in better overall survival [13,14], although this was not confirmed by other studies [15,16]. Second generation specific ABCB1 inhibitor PSC833 failed to show clinical benefit in multiple clinical trials of AML [17,18,19]. However, a study reported a minor clinical benefit of PSC833 in AML patients younger than 45 years of age [20].

In this study, we aimed to comprehensively evaluate the clinical relevance of ABCB1 activity in AML; firstly, by determining its association with clinical parameters (clinical, cytogenetic, and molecular), gene expression, and ex vivo drug resistance in patients with de novo AML. Furthermore, we examined clinical outcome in high ABCB1 activity AML patients treated with the combination of the ABCB1 inhibitor quinine with standard chemotherapy and we investigated the role of ABCB1 in AML patients treated with GO plus chemotherapy. We used global gene expression to gain new insights into why high ABCB1 maybe associated to worse clinical outcome in AML.

## 2. Results

### 2.1. Association of ABCB1 Activity with Diagnostic Parameters of AML

**Figure 1:**
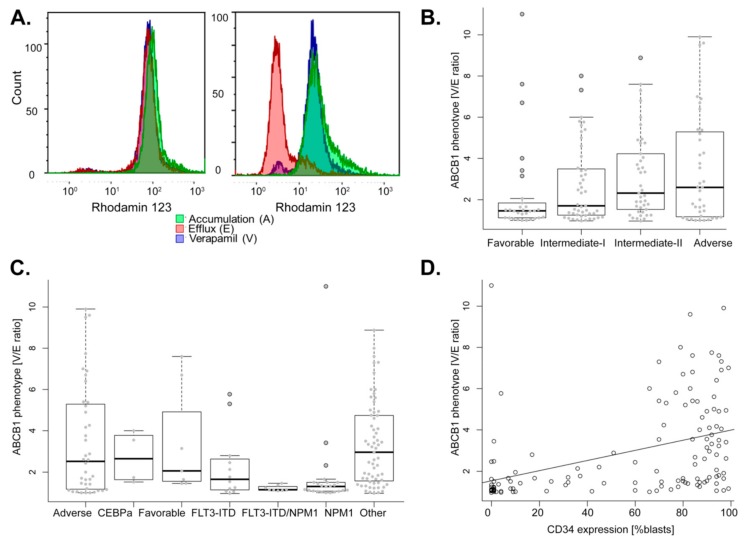
ABCB1 activity and the biological features associated with it in AML. (**A**) ABCB1 activity was determined by flow cytometry. Shown are overlay histograms of primary AML cells with a representative example of a negative ABCB1 phenotype (left panel) showing no rhodamine 123 efflux and high ABCB1 phenotype (right panel) indicating rhodamine 123 efflux with complete reversal by the ABCB1 inhibitor verapamil. (**B**) ABCB1 activity associated with ELN classification. Boxplot showing the median and the interquartile range of ABCB1 activity within the four ELN classes, outliers are indicated as open circles. (**C**) ABCB1 activity associated with molecular classes defined by caryotype, *CEBPA*, *FLT3*, and *NPM1* mutational status. Boxplot showing the median and the interquartile range of ABCB1 activity. Data points are indicated as grey points, outliers as open circles. (**D**) Scatter plot of ABCB1 activity and CD34 protein expression on AML blast cells. Linear regression line is shown (*p* < 0.001, *r* = 0.41, Pearson correlation; *p* < 0.001, *rho* = 0.51, Spearman’s rank test).

A positive ABCB1 phenotype was defined as a V/E ratio (verapamil reversal of the efflux) greater than 1.5 within the blast population and this was detected in 58% of AML (94 of 161). A representative flow cytometry analysis of an ABCB1 negative and positive phenotype is shown in Figure 1A.

In our cohort of adult de novo AML, age at diagnosis was not higher in ABCB1 positive AML compared to ABCB1 negative AML (Figure S1A, Table 1), and no relation was observed between ABCB1 phenotype and cytogenetic classification (Figure S1B). Nevertheless, ABCB1 phenotype was associated to ELN classification with low ABCB1 activity found in favorable and high ABCB1 activity found in adverse ELN classes (*p* = 0.038; Figure 1B). When compared among cytogenetic and molecular subtypes, ABCB1 activity is specifically low in AML with either mutant *NPM1* or *FLT3-ITD*, and in AML with both markers. ABCB1 activity is highly variable within AML with adverse and favorable cytogenetics as well as in AML without known prognostic genetics (Figure 1C). When focused on the mutational status of *CEBPA*, *NPM1*, and *FLT3*, these analyses demonstrated that ABCB1 positivity is associated with the absence of both *FLT3-ITD* and *NPM1* mutations (*p* = 0.006 and *p* < 0.001; Table 1). We found that positive ABCB1 activity was associated with high expression of CD34, specifically, in ABCB1 positive AML 85% of blasts express CD34 compared to ABCB1 negative AML in which only 12% of blasts express CD34 (*p* < 0.001; Table 1, Figure 1D). ABCB1 positivity was associated with low white blood cell count (WBC) median: 4.7 vs. 16.2 G/L found in ABCB1 negative AML (*p* = 0.011; Table 1).

Thus, positive ABCB1 activity was linked to adverse cytogenetics, high CD34 expression, and low WBC count. Furthermore, negative ABCB1 activity occurred in AML carrying the *NPM1* mutation.

### 2.2. Higher ABCB1 Activity Linked to Poor Clinical Outcome of AML Patients Treated with Standard Chemotherapy

**Figure 2:**
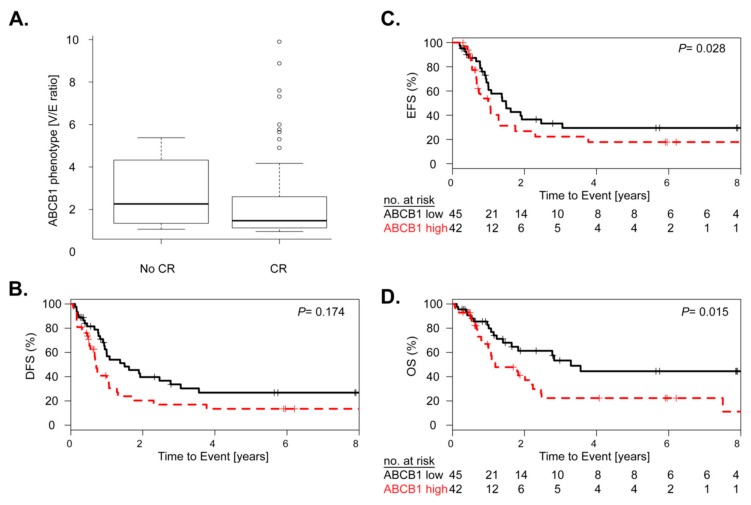
ABCB1 activity in relation to clinical response after intensive chemotherapy. (**A**) Boxplot of ABCB1 activity and response to remission induction therapy showing the median and the interquartile range in patients failing initial therapy (No CR) versus patients obtaining complete remission (CR, *p* = 0.17, MWU test). Cox proportional hazard analysis showing the association ABCB1 activity with (**B**) disease-free (DFS), (**C**) event-free survival (EFS), and (**D**) overall survival (OS) of patients with low ABCB1 (less than the median, black) versus high ABCB1 (higher than the median, red) activity. Numbers at risk at each year of follow-up are given. *p*-values based on log rank test.

CR after remission induction chemotherapy was evaluated in 87 patients and was achieved in 86% of patients. Although not significant, lower CR rates (81%) were obtained in patients with positive ABCB1 AML compared to those with negative ABCB1 activity (91%) and ABCB1 activity was higher in non-responders vs. patients in CR (median V/E ratio: 2.2 vs. 1.5, *p* = 0.17, Figure 2A). Similarly, positive ABCB1 activity was not significantly linked to disease-free survival (DFS, *p* = 0.174; Figure 2B). Of note, a conceivable reason that CR and DFS were not significantly associated to ABCB1 activity may be the smaller number of patients included (*n* = 76).

Nevertheless, positive ABCB1 activity was significantly associated with worse event-free survival (EFS, hazard ratio (HR) = 1.79, 95% confidence interval (CI) = 1.06–3.03, *p* = 0.028) and OS (HR = 2.09, 95% CI = 1.14–3.83, *p* = 0.015; Figure 2C,D). Multivariate analysis including ELN classification, WBC count at diagnosis and ABCB1 activity as covariates, established the independent negative impact of positive ABCB1 activity on EFS and on OS (HR = 1.84, 95% CI = 1.02–3.32, *p* = 0.044; HR = 1.98, 95% CI = 1.03–3.81, *p* = 0.039; Table 2).

In summary, high ABCB1 activity demonstrated an independent negative impact on event-free and overall survival of AML treated with standard chemotherapy.

### 2.3. Concurrent Treatment with the ABCB1 Inhibitor Quinine Did Not Improve Clinical Outcome

**Figure 3:**
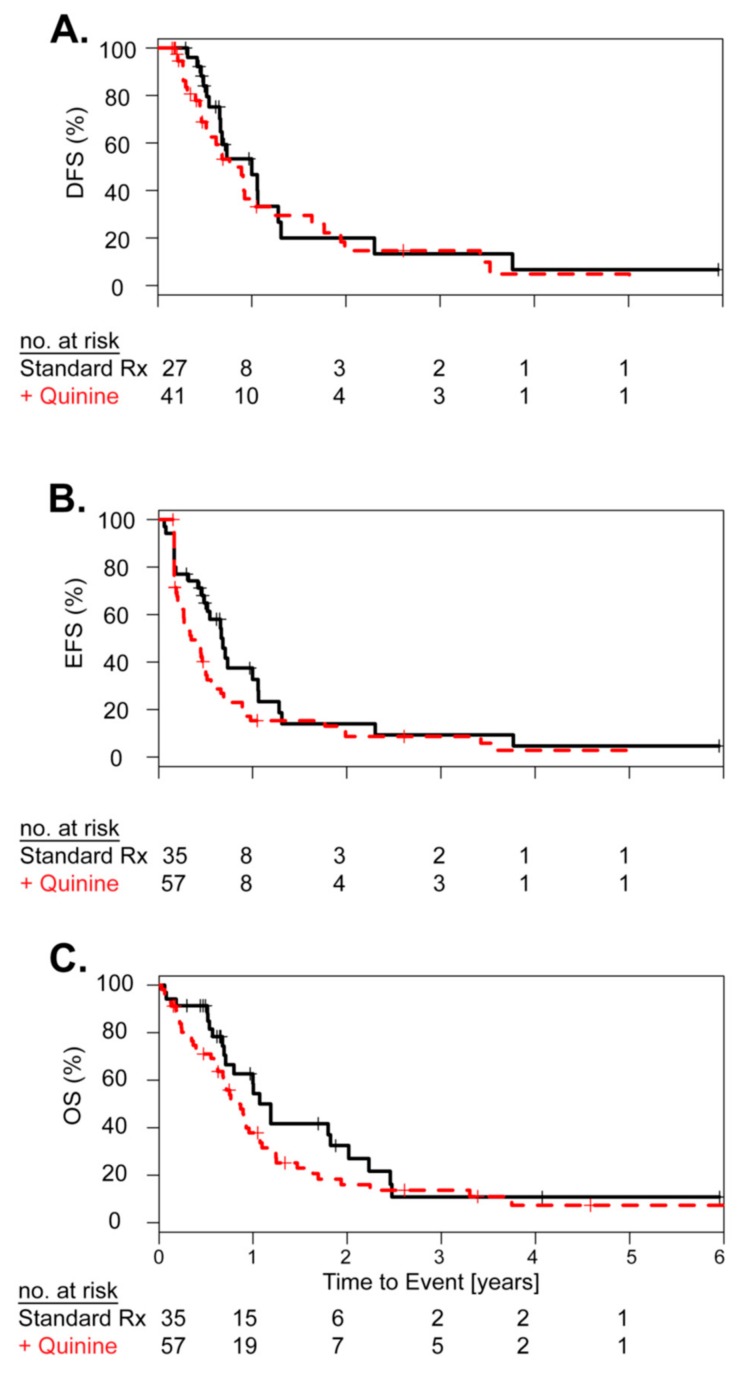
ABCB1 activity in relation to clinical response after standard chemotherapy plus quinine. Cox proportional hazard analysis showing the effect of quinine therapy on (**A**) disease-free survival (DFS), (**B**) event-free survival (EFS), (**C**) overall survival (OS) of patients with ABCB1 high AML with standard chemotherapy (black) and standard chemotherapy plus the ABCB1 inhibitor quinine (red). Numbers at risk at each year of follow-up are given. *p*-values are based on log rank test.

A small pilot cohort of patients with high risk AML and positive ABCB1 activity was treated with the first generation ABCB1 inhibitor quinine (*n* = 57). The median age was 57 years (range: 37–78). Toxicities related to the ABCB1 inhibitor quinine occurred in the majority of patients and included nausea and vomiting (mild 75%, severe 11%), mild tinnitus (77%), diarrhea (54% mild, 9% severe), and mucositis (58% mild, 9% severe). As this was a single arm pilot study, we used a subset of 35 of the 161 AML patients with positive ABCB1 activity, treated with standard chemotherapy matched for cytogenetics and molecular markers as a control cohort for outcome comparison. The median age was 56 years (range: 22–76). In the standard chemotherapy plus quinine pilot cohort 70% achieved CR, compared to 77% in the matched standard chemotherapy only control cohort. Neither CR rate, DFS, EFS, nor OS were significantly different when comparing standard chemotherapy plus quinine vs. standard chemotherapy alone (Figure 3A–C). In in this pilot study, we found no significant clinical advantage for the addition of the ABCB1 inhibitor quinine to standard chemotherapy and quinine related toxicities were frequent and sometimes severe (about 10%).

### 2.4. Primary Cell ABCB1 Activity Was Not Linked to Ex Vivo Drug Resistance and Showed a Specific Gene Expression Profile

**Figure 4:**
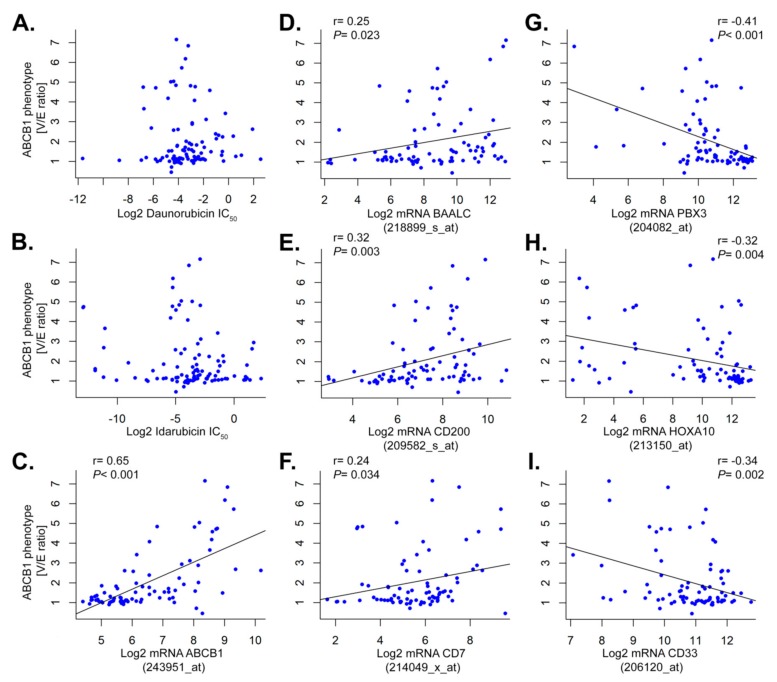
ABCB1 activity in relation to drug resistance and correlated gene expression. Scatterplots showing ABCB1 activity in relation to (**A**) Daunorubicin and (**B**) Idarubicin resistance. Resistance is represented as IC_50_ no significant correlation was detected. ABCB1 activity was highly associated with its transcriptional level (*p* < 0.001, *r* = 0.64, Pearson correlation; *p* < 0.001, *rho* = 0.62, Spearman’s rank test) (**C**). Other genes correlated positively with ABCB1 activity were (**D**) BAALC (*p* = 0.023, *r* = 0.25, Pearson correlation; *p* = 0.011, *rho* = 0.28, Spearman’s rank test); (**E**) *CD200* (*p* = 0.003, *r* = 0.32, Pearson correlation; *p* < 0.001, *rho* = 0.39, Spearman’s rank test); and (**F**) *CD7* (*p* = 0.034, *r* = 0.24, Pearson correlation; *p* = 0.020, *rho* = 0.26, Spearman’s rank test), on the contrary genes negatively correlated were (**G**) *PBX3* (*p* < 0.001, *r* = −0.42, Pearson correlation; *p* < 0.001, *rho* = −0.43, Spearman’s rank test); (**H**) *HOXA10* (*p* = 0.004, *r*= −0.32, Pearson correlation; *p* < 0.001, *rho* = −0.41, Spearman’s rank test); and (**I**) *CD33* (*p* = 0.002, *r* = −0.34, Pearson correlation; *p* = 0.022, *rho* = −0.25, Spearman’s rank test) with respective probe set identifiers in parenthesis. For significant correlations linear regression lines are shown; *r* and *p*-values are based on Pearson correlation.

In an attempt to explain the clinical inefficiency of ABCB1 inhibition, we evaluated the relationship between drug resistance and ABCB1 activity. From the same primary patient sample, we obtained ABCB1 phenotype, gene expression, and ex vivo drug resistance to the anthracyclines daunorubicin and idarubicin, both drugs are reported substrates of the ABCB1 transporter. We showed that high ABCB1 activity was not correlated to neither daunorubicin nor idarubicin ex vivo drug resistance (Figure 4A,B).

As ABCB1 activity was highly correlated with its mRNA expression (*r* = 0.64, *p* < 0.001; Figure 4C), we used mRNA expression as a substitute for ABCB1 activity to gain further mechanistic insights. Firstly, we aimed to identify co-regulated genes and found *BAALC*, *CD200*, and *CD7* positively correlated with ABCB1 expression (*r* = 0.25, *p* = 0.023; *r* = 0.32, *p* = 0.034; *r* = 0.24, *p* = 0.010; Figure 4D–F). On the contrary, *PBX3*, *HOXA10*, and *CD33* were negatively correlated to ABCB1 (*r* = −0.42, *p* < 0.001; *r* = −0.32, *p* = 0.004; *r* = −0.34, *p* = 0.002; Figure 4G–I).

**Figure 5:**
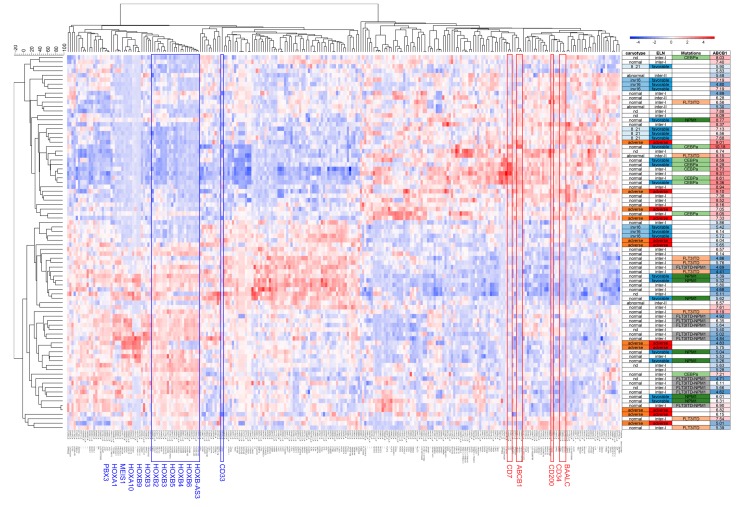
ABCB1 activity in relation to drug resistance and co-regulated gene expression. Hierarchical clustering representing genes in columns AML samples in rows. The dendrogram outlines two main clusters of patients based on *ABCB1* expression illustrated in the row annotation using a color scale of low to high (blue to red). Furthermore, ELN groupings are indicated as favorable (blue) and adverse (red); and mutations such as *NPM1* (dark green), *CEBPA* (light green), *FLT3-ITD* (salmon), *FLT3-ITD + NPM1* (grey).

Secondly, we performed hierarchical clustering on 255 gene probes most highly positively or negatively correlated with ABCB1 activity (i.e., *p* < 0.0001; false discovery rate (FDR) < 2% and absolute *r* > 0.3) (Figure 5). We observed two clusters with mainly positive vs. negative ABCB1 activity AML. Negative ABCB1 AML were more frequently mutant *NPM1* with low *CD34* expression and high expression of *HOX* cluster genes. In line with respective poor clinical outcome, positive ABCB1 AML overexpressed *CD34*, *BAALC*, and *CD200*. Apart from visualizing co-regulated gene expression, this analysis gave further insights into the inherent heterogeneity of AML with regard to ABCB1. For example, not all adverse AML were ABCB1 high, not all mutant *NPM1* AML were ABCB1 low and a majority of *CEBPA* mutant AML were ABCB1 high despite *CEBPa* being a favorable prognostic marker. Finally, *CD33* was negatively correlated with ABCB1 for both mRNA and activity levels (*r* = −0.34, *p* = 0.002, Figure 4I; *r* = −0.28, *p* = 0.002, Supplemental Figure S2).

We found no significant association between ABCB1 activity and ex vivo drug resistance in primary AML blast cells. However, correlative gene expression analysis pointed to new insights as to why ABCB1 may be linked to poor prognosis.

### 2.5. Patients with Low ABCB1 Expression Benefit from Addition Ofgemtuzumab Ozogamicin (GO) to Standard Chemotherapy

**Figure 6:**
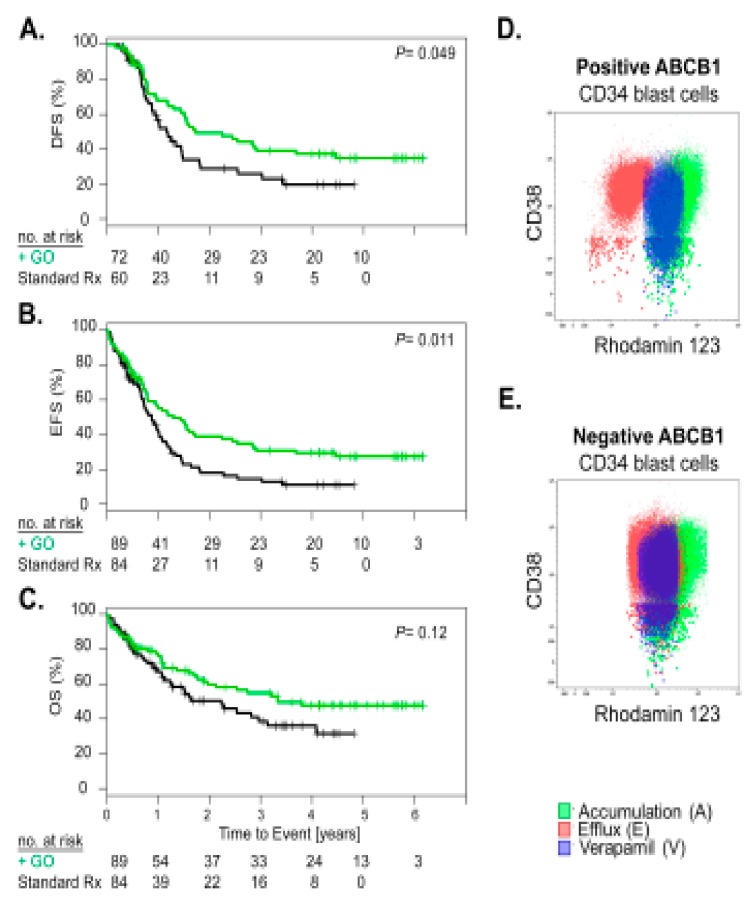
Lower ABCB1 activity AML benefits from GO addition to standard chemotherapy and total blast ABCB1 activity is reflected by its CD34^+^CD38^−^ stem cell ABCB1 activity. Cox proportional hazard analysis demonstrating a beneficial effect of GO addition to standard chemotherapy (**A**) on disease-free survival (DFS), (**B**) on event-free survival (EFS) and (**C**) on overall survival (OS) of patients with lower ABCB1 expression with the standard chemotherapy stratification in black and standard chemotherapy plus GO stratification in green. Numbers at risk at each year of follow-up are given. *p*-values based on log rank test. Dot plots of rhodamine 123 accumulation, efflux, and verapamil reversal on CD34+ blast cells showing that ABCB1 positive (**D**) and negative (**E**) AML total blasts and the subpopulation of CD34+CD38− stem cells have a similar phenotype.

The cell surface protein CD33 is targeted by the anti-CD33 drug conjugate gemtuzumab ozogamicin (GO). Because ABCB1 was negatively correlated to CD33 expression, we examined whether his had any effect on clinical response to GO. Among the 194 patients of the ALFA-0701 trial, 173 patients had low to intermediate *ABCB1* expression and 21 had high *ABCB1* expression. In patients with lower *ABCB1* expression, addition of GO to standard chemotherapy was associated with significantly better DFS (*p* = 0.049) and EFS (*p* = 0.011) (Figure 6A,B). However, there was only a trend for a better OS (*p* = 0.118) (Figure 6C). Conversely, addition of GO showed no significant impact on survival in patients with high *ABCB1* expression (Figure S2D–F). We showed that *ABCB1* expression maybe useful to guide selection of GO responsive AML cases.

### 2.6. ABCB1 Expression Is Related to the Stem Cell Phenotype

Because ABCB1 was highly associated with CD34 and CD33 we postulated that ABCB1 may be a bystander effect inherent to a respective leukemia stem cell (LSC) phenotype. For some samples, we performed the CD34 and CD38 staining in combination with ABCB1 activity. We found that if the progenitor population defined as CD34+ and CD38− was ABCB1 positive the total blast population was also ABCB1 positive and vice versa (Figure 6D,E). Furthermore, ABCB1 activity was not significantly related to the LSC17 gene signature (data not shown) [21], indicating that positive ABCB1 activity may not be specific to the LSC but also relates to normal hematopoietic stem cells and that positive ABCB1 activity may not be a specific marker of aggressiveness of the leukemia cells. Therefore, drug resistance by drug efflux may not be the primary mechanism explaining poor outcome of ABCB1 positive AML.

We found that ABCB1 expression of the leukemia bulk is related to its hematopoietic progenitor cell ABCB1 expression.

## 3. Discussion

Identification of new prognostic markers, remains important and only three molecular markers (NPM1 and CEBPA mutations, FLT3-ITD) are currently used in clinical practice [2]. Here, we evaluated the clinical characteristics of the ABCB1 phenotype and its role in clinical outcome of AML using the specific ABCB1 functional assay with rhodamine 123 and verapamil.

In our study in adult AML patients treated with standard chemotherapy, the median ABCB1 activity was not significantly higher in AML patients not achieving CR after remission induction therapy. Similarly, disease relapse did not occur more frequently in positive ABCB1 AML. This finding indicates that apart from drug resistance to chemotherapy other co-operative mechanisms likely exist explaining the negative effect of positive ABCB1 activity on long-term treatment outcome of AML. It has been reported that multiple transporters of the ABC family may confer resistance to chemotherapy, among them ABCB1, ABCC1, and ABCG2 have been described most frequently [3,4]. Of note, we cannot rule out effects by ABCB1 single nucleotide polymorphism (SNP), even though mutations in ABCB1 in AML are rare (www.cbioportal.org).

Yet, ex vivo drug resistance testing of primary AML cells revealed no correlation between high ABCB1 activity and neither daunorubicin nor idarubicin resistance. In addition, the impact of ABCB1 activity and expression was more prominent on later clinical events like OS and EFS and was neither significant for CR nor DFS. In particular, as inherent drug resistance is thought to rather impact the latter two. This would explain why most associations between ABC transporters and clinical outcome are correlative and have not been shown to be causal [3,4]. Nevertheless, our larger study confirms the independent negative impact of high ABCB1 phenotype on AML clinical outcome in particular on EFS and OS. Furthermore, we confirmed the association of high ABCB1 phenotype with increased expression of CD34 [22,23,24], a characteristic marker of the immature subset in AML blast cells. However, we newly identified that high ABCB1 activity was significantly associated with low WBC count and absence of *FLT3-ITD* and *NPM1* mutations and this was not described in previous studies where mutational status of these genes was available [22]. These authors used the JC1 assay to assess ABC efflux activity and pointed out that this probe is probably not ABCB1 specific. In our study, we used a specific test for ABCB1 activity (rhodamine 123 plus verapamil) and thus independently confirmed in a larger cohort of AML that ABCB1 activity and expression confers poor outcome in AML. We also show that the worse prognosis of ABCB1 activity was independent of ELN classification. Given the important role of prognostic markers for treatment management, ABCB1 activity may represent a possible new marker in patients lacking known mutations to guide therapy (i.e., treatment intensification or allogeneic stem cell transplantation).

Since newer generation and more selective ABCB1 inhibitors have failed to prove clinical relevance, it was suggested that possibly larger spectrum ABC efflux transporters maybe more effective as multiple efflux transporters maybe involved to form the MDR phenotype [4]. To assess if ABCB1-targeted therapy would be efficient to sensitize AML to chemotherapy, we performed a clinical trial with quinine which is a first generation ABCB1 inhibitor. Even though our pilot study (idarubicin) did not include a designed control arm, the analysis with a control group (daunorubicin) indicated that quinine treatment did not significantly improve survival which is in line with two other controlled phase III studies [15,16]. Furthermore, toxicity remains a major concern with significant off-target effects like we have described above.

We then hypothesized that evaluation of ABCB1 co-expressed genes may uncover potential mechanisms explaining how high ABCB1 leads to treatment failure in AML. Our transcriptomic analysis revealed a positive correlation among *ABCB1*, *CD200*, and *BAALC*. Similar to *ABCB1*, the latter two had also been associated with an adverse prognosis in AML [4,22,23,25,26]. However, overexpression of *BAALC* was not associated in AML with high ABCB1 activity in another study, probably due to its restriction to CN-AML [22]. More interestingly, *CD200* was involved in the development of an impaired immune system in response to AML [27]. This protein was associated with worse OS when upregulated in AML and weak expression CD200 was related to AML responding well to remission induction chemotherapy [26].

Our transcriptomic analysis showed that *CD33* was negatively correlated with *ABCB1* expression. CD33 is expressed on myeloid progenitors and is the therapeutic target of the drug GO. Treatment with GO was beneficial in terms of DFS and EFS for patients with lower *ABCB1* expression. Our findings are in line with previous studies showing high ABCB1 activity was associated with a poorer response to GO [28] and that CD33 expression was negatively correlated to ABCB1 efflux [11]. Notably, direct experimental evidence linking GO resistance to its drug efflux via ABCB1 in primary AML is still lacking. Nevertheless, our results imply that assessing the level of ABCB1 expression may be important as GO addition showed clinical benefit in low ABCB1 expressing AML. Furthermore, for a subset of AML we determined ABCB1 activity and CD34/CD38 staining showing that the MDR phenotype is associated to a hematopoietic stem cell phenotype as it is known that the expression of ABCB1 correlates with the expression of CD34, a characteristic marker of the immature subset in AML blast cells [24]. Further studies are necessary to better understand the specific relation between ABC transporters and stem cell phenotypes.

## 4. Materials and Methods

### 4.1. Patients’ Samples

Patients were classified according to ELN 2010 by cytogenetics and molecular markers into favorable: t(8;21), inv(16), normal karyotype/mutant NPM1/wild-type FLT3, normal karyotype/mutant CEBPA; adverse: inv(3), t(6;9), −5, −7, abnormal 17p, complex, MLL rearranged; intermediate—II: t(9;11), other non-normal karyotypes; intermediate—I: normal karyotype/mutant NPM1/FLT3-ITD, normal karyotype/wild-type NPM1/FLT3-ITD, other normal karyotypes.

ABCB1 activity was measured in a cohort of 161 adult AML patients, diagnosed and treated at the Lille Hospital between 2000 and 2010, their median age was 60.7 years (range: 21–88). Clinical features are summarized in Table 1. Of those, 87 were treated with standard intensive chemotherapy (cytarabine/daunorubicin) and 74 were not treated with standard chemotherapy (i.e., low-lose cytarabine, azacytidine, or palliative therapy), the latter group of patients was excluded from survival analyses.

The single arm quinine pilot study included 57 AML patients recruited at the local Lille Hospital between 2000 and 2008 and treated with intensive chemotherapy (cytarabine/idarubicin) plus the ABCB1 inhibitor quinine. The clinical protocol MAquinine was approved by the local hospital review board. Inclusion criteria were high ABCB1 activity, non-favorable disease characteristics, and absence of any signs of heart failure or abnormal kidney or liver function. Median age was 57.0 (range: 37–78). Quinine treatment was administrated as previously described [15].

For ABCB1 activity, *ABCB1* gene expression and ex vivo drug resistance were performed in 103 adults diagnosed with de novo AML at Lille Hospital between 2010 and 2014. The median age was 55.4 years (range: 20–82). Finally, we included a subset of 194 AML patients treated with or without GO plus chemotherapy from the ALFA 0701 clinical trial for which *ABCB1* gene expression data was available [21,29]. Median age was 62.3 years (range: 50.2–70.9).

All studies were approved by the local ethics committee and informed consent was obtained at diagnosis. A CONSORT diagram outlines the different cohorts of patients and associated datatypes (Supplementary Materials).

All patient studies were approved by the local ethics committee and informed consent was obtained at diagnosis.

The local ethics committee approved all studies and all patients signed an informed consent at diagnosis (CHRU de Lille, Tumorothèque du C2RC, approval nos. CSTMT079, CSTMT089).

### 4.2. ABCB1 Activity

ABCB1 activity was measured by multiparametric flow cytometry (MFC). Blast cells were washed twice at 37 °C with RPMI-1640 supplemented with 10% fetal bovine serum (RPMI-FBS) (Thermo Fisher, Waltham, Massachusetts, Biowest, Wellington, CO, USA). Firstly, accumulation (A) of rhodamine-123 (rh123, Sigma-Aldrich, Saint Louis, MI, USA), in the cells was determined by exposing 1.5 × 10^6^ cells to 0.2 µg/mL rhodamine 123 for 45 min in the dark at room temperature. The cells were then washed twice with RPMI-FBS at 4 °C. Secondly, evaluation of the rhodamine 123 efflux (E) was performed after a 2-h incubation at 37 °C with and without 10 µg/mL Verapamil (V, Mylan, Canonsburg, PA, USA). The cells were washed twice with RPMI-FBS at 4 °C and stained with anti-CD45-PC5.5 (clone J33, Iotest, Beckman Coulter, Brea, CA, USA) for 30 min in the dark at room temperature, washed twice, and analyzed by MFC. Blast cells were gated as CD45^dim^ and SSC^low^. Two ratios of mean fluorescence intensities were calculated: E/A (rhodamine 123 efflux) and V/E (verapamil reversal of the efflux). Experiments were performed on a Navios flow cytometer and analyzed with Kaluza software (Beckman-Coulter). The cytometer settings were tested daily for optical alignment, fluidic stability, and optical detector sensitivity using standard fluorospheres (Flowset targets™ and Flowcheck™, Beckman Coulter, Villlepinte, France).

Five patients were analyzed with an anti-CD34-AA700 (clone 581, Iotest, Beckman Coulter) and an anti-CD38-PB (clone LS-198-4-3, Iotest, Beckman Coulter, Villepinte, France) simultaneously with the measurement of ABCB1 activity to evaluate the CD34+CD38- population known to be enriched for leukemia stem cells.

### 4.3. Gene Expression Analysis

Gene expression analysis was performed on Affymetrix GeneChip™ U133 Plus 2.0 array according to the manufacturer’s directions (Thermo Fisher), using total RNA extracted from cryopreserved AML samples and log2 gene expression values were obtained as described previously [30]. We filtered out probe sets if they were present in less than 10% of samples (absence filtering) and if standard deviation was less than σ <1.1 (non-variance filtering). All gene expression data will be made available via GEO at https://www.ncbi.nlm.nih.gov/geo/.

### 4.4. Ex Vivo Drug Resistance

The MTT (3-4,5-dimethylthiazol-2-yl-2.5-difenyl tetrazolium bromide) assay was used to study drug resistance in conditions as previously described [31]. The following drugs and concentrations were used: daunorubicin (0.00004–3.33 µg/mL) and idarubicin (0.000026–2 µg/mL). The cytotoxicity was expressed as the inhibitory concentration to 50% of the cells (IC_50_).

### 4.5. Statistical Analysis

The association of the MDR phenotype with biological factors was assessed with Student t- or Mann–Whitney U (MWU) test and qualitative variables were compared using Chi-square or Fisher exact test. Outcome analysis was performed as previously described [31]. Time was censored at transplantation date in the event of allogeneic hematopoietic stem cell transplantation. OS was defined as the time from date of inclusion to date of death from any cause or time was censored at date of last known alive. EFS was defined as the time from date of inclusion to date of first event (i.e., refractory disease, relapse, or death from any cause) or in the cases of no event, time was censored at the date of last follow up. DFS was determined only in patients achieving CR and is defined as the time from date of CR to date of first relapse or in the case of no relapse, time was censored at the date of last follow up. All statistical tests were performed with R version 3.5.2 software (R Development Core Team, Vienna, Austria).

## 5. Conclusions

We showed that ABCB1 activity and expression was linked to worse prognosis and therefore evaluating its activity or expression at diagnosis may still be important. However, ABCB1 did not directly mediate chemotherapy resistance ex vivo and in vivo and therefore we hypothesize that ABCB1 may be a bystander effect. We provide several potential candidate genes associated with high ABCB1 which are directly involved in hematopoiesis or affect immunologic response. Finally, ABCB1 activity had an independent prognostic role and was not solely associated with *FLT3-ITD* and/or *NPM1* mutational status. Thus, assessment of the ABCB1 phenotype may help to refine AML treatment as GO may not be effective in AML when ABCB1 expression is high.

## Figures and Tables

**Table 1:** Patient characteristics.

	Positive ABCB1 *n* = 94 (58%)	Negative ABCB1 *n* = 67 (42%)	*p*-Value
**Median Age** in years (range)	60.6 (22.3–86.7)	60.7 (21.3–87.9)	0.99
**Median WBC count** as 10^9^/L (range)	4.7 (0.8–208.0)	16.2 (0.7–378.0)	0.011
**FAB subtypes***n* (%)			
M0	8 (8)	4 (6)	<0.001
M1	11 (12)	15(22)	
M2	31 (33)	8 (12)	
M4	8 (9)	13 (19)	
M5	3 (3)	15 (22)	
M6	2 (2)	0	
M7	2 (2)	0	
ND	29 (31)	12 (18)	
**Cytogenetics**			
Favorable	6 (6)	2 (3)	0.556
Intermediate	62 (66)	49 (73)	
Adverse	26 (28)	16 (24)	
**Median CD34 expression** as % of blasts (range)	85.0 (0.0–99.0)	12.0 (0.0–99.0)	<0.001
**NPM1 mutation***n* (%)			
Present	4 (4)	29 (43)	<0.001
Absent	75 (80)	32 (48)	
ND	15 (16)	6 (9)	
**FLT3-ITD***n* (%)			
Present	7 (7)	16 (24)	0.006
Absent	77 (82)	47 (70)	
ND	10 (11)	4 (6)	
**CEBPA mutation ***n* (%)			
Present	6 (6)	0 (0)	0.037
Absent	73 (78)	60 (90)	
ND	15 (16)	7 (10)	

WBC: white blood cell; ABCB1: P-glycoprotein; ND: not done.

**Table 2:** Multivariate analysis

Characteristics	DFS HR	95% CI	*p*-Value	EFS HR	95% CI	*p*-Value	OS HR	95% CI	*p*-Value
**ELN classification**									
Favorable	-	-	-	-	-	-	-	-	-
Intermediate-I	2.1	0.89–4.94	0.091	2.6	1.15–5.89	**0.022**	2.34	0.90–6.31	0.082
Intermediate-II	3.71	1.48–9.31	**0.005**	4.5	1.88–10.7	**0.007**	4.5	1.72–11.8	**0.002**
Adverse	13.9	4.02–48.1	**<0.001**	12.6	4.40–35.9	**<0.001**	19.9	5.91–67.2	**<0.001**
**WBC count**									
<100	-	-	-	-	-	-	-	-	-
>100	4.22	1.32–13.5	**0.015**	2.95	1.16–7.49	**0.023**	3.68	1.40–9.72	**0.008**
**ABCB1 activity**									
Low	-	-	-	-	-	-	-	-	-
High	1.66	0.84–3.28	0.146	1.84	1.02–3.32	**0.044**	1.98	1.03–3.81	**0.039**

ELN: European Leukemia Net, WBC: white blood cell, ABCB1: P-glycoprotein, *p*-value based on logrank test *p* < 0.05, DFS: disease-free survival, EFS: event-free survival, OS: overall survival, HR: hazard ratio, CI: confidence interval.

## Supplementary Materials

The following are available online at https://www.mdpi.com/2072-6694/11/9/1323/s1, CONSORT diagram, Figure S1: ABCB1 phenotype in relation to age at diagnosis and cytogenetics, Figure S2: ABCB1 phenotype in relation to CD33 expression and clinical outcome in high ABCB1 AML treated with or without GO addition, and Figure S3: ABCB1 activity was determined by flow cytometry (MFC).
