# Supplementary material for: Clinical Significance of ABCB1 in Acute Myeloid Leukemia: A Comprehensive Study
Source: Cancers (Basel). 2019 Sep 6;11(9):1323. doi: 10.3390/cancers11091323 (PMC6770064; doi:10.3390/cancers11091323)
Supplement: Supplementary file 1 [file cancers-11-01323-s001.pdf]

CONSORT diagram

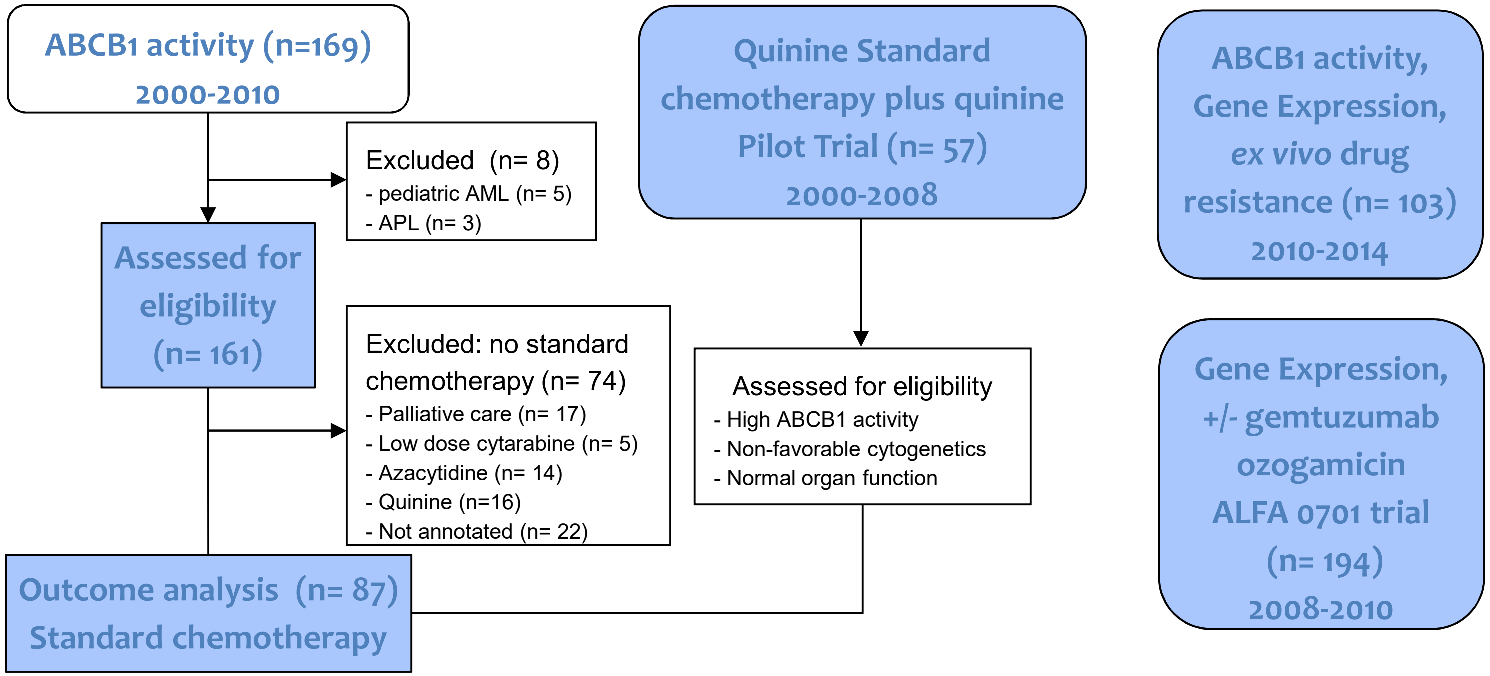

Fig. S1

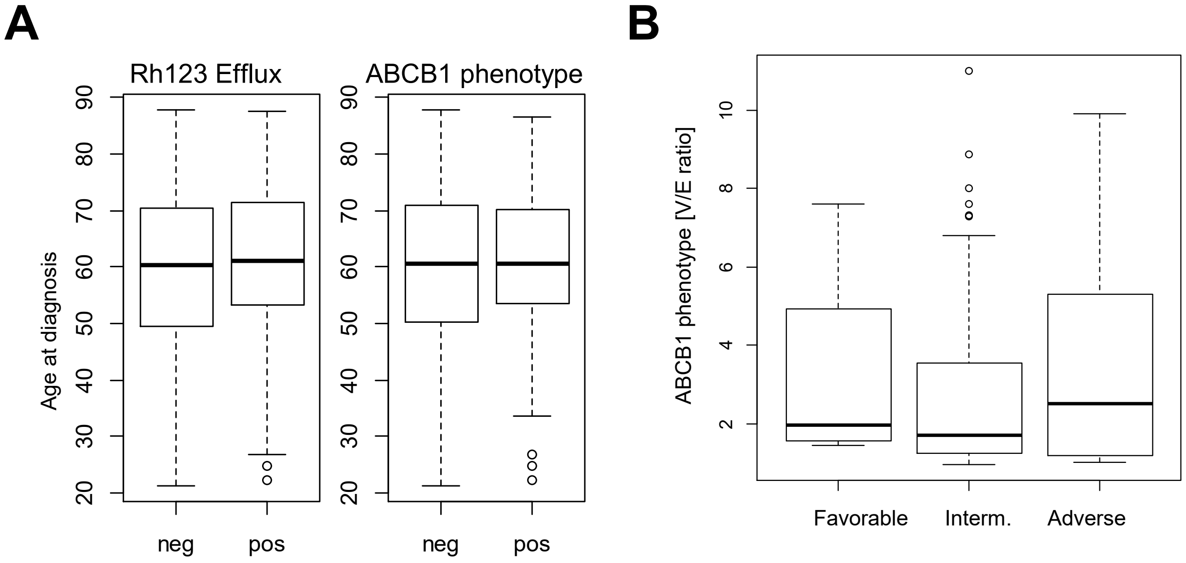

Figure S1:

ABCB1 phenotype in relation to age at diagnosis and cytogenetics. (**A**) Neither rhodamine123 efflux nor the specific ABCB1 phenotype were related to age in our adult AML cohort. (**B**) ABCB1 activity was not significantly related to AML cytogenetics.

Figure S2:

ABCB1 phenotype in relation to CD33 expression and clinical outcome in high ABCB1 AML treated with or without GO addition. (**A**) Boxplot showing lower %blasts CD33 expression in positive ABCB1 activity AML. (**B**) Accordingly, %blasts CD33 expression is negatively correlated with ABCB1 activity . Furthermore, the negative correlation of protein expression extends to the transcript level (**C**).

Cox proportional hazard analysis demonstrating no beneficial effect of GO addition to intensive chemotherapy (**D**) on disease-free survival (DFS), (**E**) on event-free survival (EFS) and (**F**) on overall survival (OS) of patients with very high ABCB1 expression with the standard chemotherapy stratification in black and standard chemotherapy plus GO stratification in green.

Fig. S2

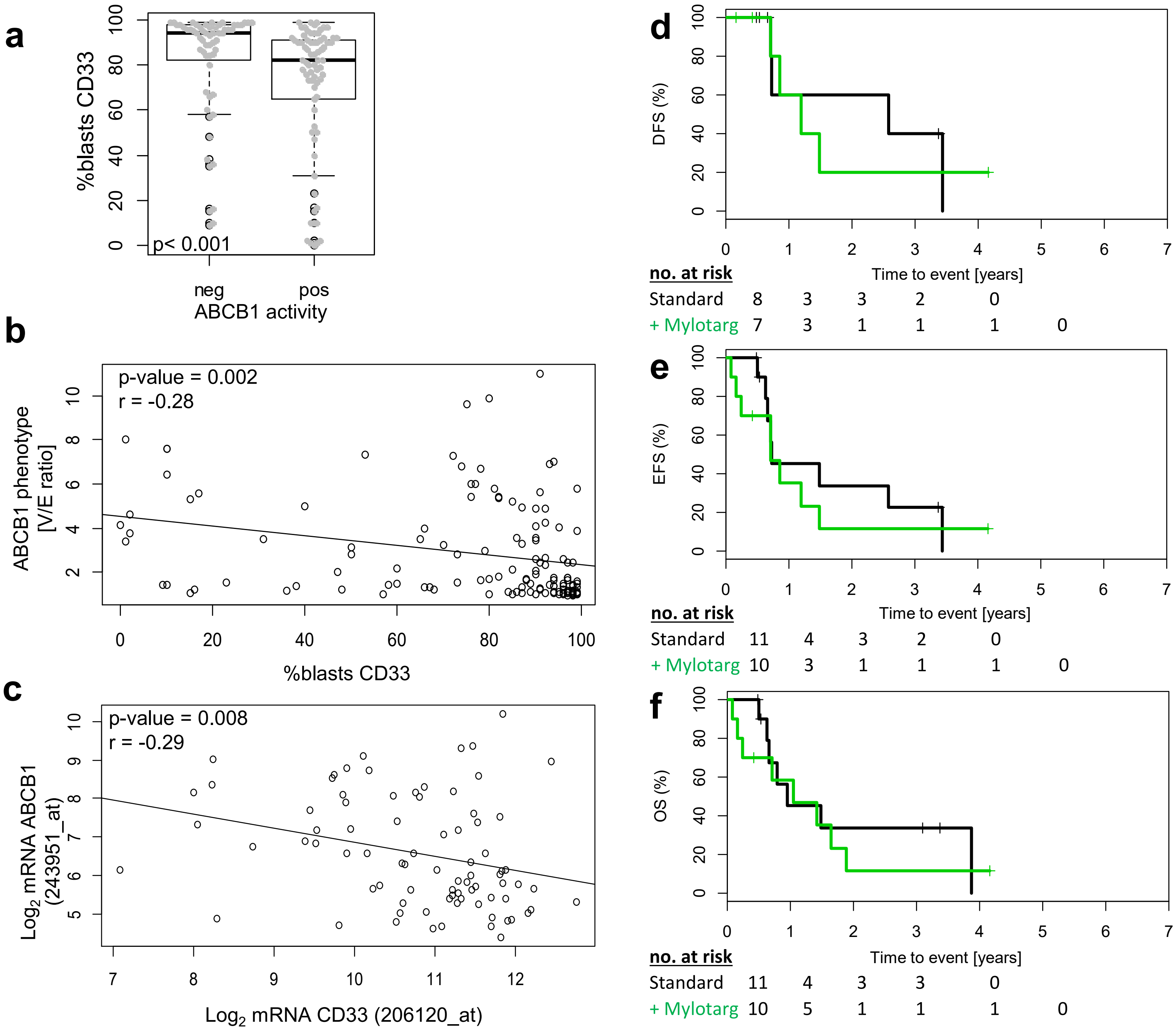

a
%blasts CD33
p< 0.001
neg
pos
ABCB1 activity
b
p-value = 0.002
r = -0.28
ABCB1 phenotype [V/E ratio]
%blasts CD33
c
p-value = 0.008
r = -0.29
Log2 mRNA ABCB1 (243951_at)
Log2 mRNA CD33 (206120_at)
d
DFS (%)
Time to event [years]
no. at risk
Standard 8 3 3 2 0
+ Mylotarg 7 3 1 1 1 0
e
EFS (%)
Time to event [years]
no. at risk
Standard 11 4 3 2 0
+ Mylotarg 10 3 1 1 1 0
f
OS (%)
Time to event [years]
no. at risk
Standard 11 4 3 3 0
+ Mylotarg 10 5 1 1 1 0

Fig. S3

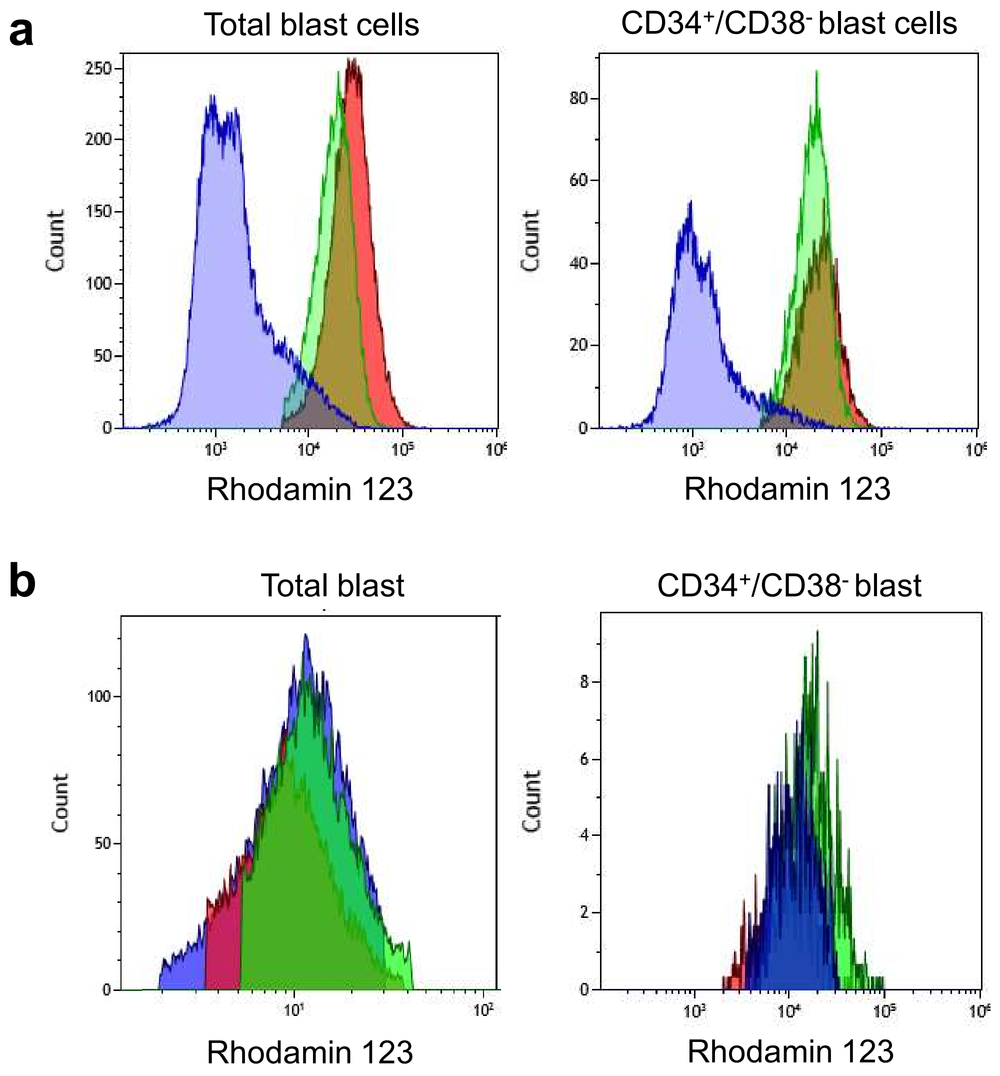

Figure S3

ABCB1 activity was determined by flow cytometry (MFC). Shown are overlay histograms of primary AML cells with a representative example of a high ABCB1 phenotype (**A**) indicating rhodamine 123 efflux with complete reversal by the ABCB1 inhibitor verapamiland a negative ABCB1 phenotype (I**B**) showing no rhodamine 123 efflux. For both cases we show total blasts and the subset of $CD34^{+}CD38^{-}$ stem cells.
